# Heart Failure Is Associated with Limb-Related Adverse Events in Patients Hospitalized for Diabetic Foot Ulcers

**DOI:** 10.3390/jcm15208024

**Published:** 2026-10-16

**Authors:** Federico Rolando Bonanni, Alessandro Minasi, Aikaterini Andreadi, Daniele Morosetti, Alfonso Bellia, Luigi Uccioli, Davide Lauro, Marco Meloni

**Affiliations:** 1 Section of Endocrinology and Metabolic Diseases, Department of Systems Medicine, University of Rome Tor Vergata, 00133 Rome, Italy; 2 Division of Endocrinology and Diabetology, Department of Medical Sciences, University Hospital Policlinico Tor Vergata, 00133 Rome, Italy; 3 Interventional Radiology Unit, Department of Biomedicine and Prevention, University of Rome Tor Vergata, 00133 Rome, Italy

**Keywords:** heart failure, diabetic foot ulcer, peripheral arterial disease, recurrent limb ischaemia, limb-related outcomes

## Abstract

**Objectives:** To evaluate the impact of heart failure (HF) on one-year outcomes in patients hospitalized for diabetic foot ulcers (DFUs). **Methods:** A retrospective observational study including consecutive patients admitted in 2024 to a tertiary multidisciplinary diabetic foot service for DFUs. Patients were stratified according to the presence of HF (HF+ vs. HF−). One-year outcomes included wound healing, major amputation, non-fatal major adverse limb events (NF-MALCE), including myocardial infarction, stroke, recurrent chronic limb-threatening ischaemia (CLTI) requiring revascularization, and mortality. **Results:** Overall, 270 patients were included (104 HF+ [38.5%] vs. 166 HF− [61.5%]). Patients with HF exhibited a more severe cardiorenal phenotype, with worse cardiac and renal biomarkers. At one-year follow-up, patients with HF showed lower wound healing rates than patients without HF (58.6% vs. 71.7%, *p* = 0.03), higher incidence of NF-MALCE (43.3% vs. 18.7%, *p* < 0.001), mainly driven by recurrent CLTI requiring revascularization (48.9% vs. 27.0%, *p* < 0.001), and higher mortality (21.1% vs. 7.2%, *p* = 0.001). Conversely, no differences were observed in major amputation, myocardial infarction, or stroke. After multivariable adjustment for age, sex, ischaemic heart disease, atrial fibrillation, dialysis, haemoglobin, albumin, foot infection and peripheral arterial disease (PAD), HF remained independently associated with NF-MALCE (adjusted odds ratio (aOR) 2.90, 95% confidence interval (CI) 1.38–6.09, *p* = 0.005) and recurrent CLTI requiring revascularization (aOR 2.43, 95% CI 1.11–5.32, *p* = 0.026); the association remained consistent in the subgroup with PAD. **Conclusions:** HF identifies a high-risk cardio-renal phenotype and is independently associated with increased limb-related adverse events in patients hospitalized for DFUs, supporting closer vascular surveillance and integrated multidisciplinary cardio-renal-foot care.

## 1. Introduction

Diabetes mellitus represents a growing global health challenge, affecting an estimated 589 million adults worldwide in 2024, with this number projected to reach 853 million by 2050 [1]. Both diabetic foot ulcers (DFUs) and heart failure (HF) represent common and clinically relevant complications in diabetic patients and are associated with a substantial clinical burden [2,3,4,5]. A significant association between DFUs and cardiovascular morbidity and mortality is well established, with an estimated prevalence of HF of approximately 25% among patients with DFUs [6].

Recently, a large study including 4937 patients with diabetes and HF demonstrated that the presence of DFUs was an independent predictor of 5-year mortality [7]. Conversely, evidence on the prognostic impact of HF in patients with DFUs remains limited, with only a few heterogeneous studies available, differing in study populations, sample sizes, and follow-up duration.

The landmark multicenter EURODIALE study found that HF was more prevalent among patients with DFUs complicated by peripheral arterial disease (PAD) and was associated with non-healing at 1 year [8]. This finding was subsequently confirmed in a dedicated study, in which HF was an independent predictor of both non-healing and delayed DFU healing at 3 months [9]. Specifically, among patients with neuro-ischaemic DFUs, HF was confirmed to be more prevalent and was also identified as an independent predictor of non-healing [10] and 1-year mortality [11,12].

Available evidence has mainly focused on healing, amputation, and mortality, whereas the impact of HF on cardiovascular and limb-related events in patients with DFUs remains less well characterized. Moreover, most available data on HF in this population derive from broader cohorts rather than specifically from patients hospitalized for DFUs. Therefore, this study aimed to evaluate one-year outcomes according to HF status in patients hospitalized for DFUs, focusing on both traditional endpoints and non-fatal major adverse limb events (NF-MALCE), including myocardial infarction, stroke, and recurrent limb ischaemia requiring revascularization.

## 2. Materials and Methods

### 2.1. Study Design and Patient Selection

This retrospective observational study included consecutive patients admitted to a specialized diabetic foot service (DiFoSe) between January 2024 and January 2025. Patients were hospitalized for DFUs complicated by limb ischaemia, infection, or other conditions requiring surgical treatment. Patients with an indication for primary amputation due to the absence of a feasible surgical option for foot salvage were excluded (Table 1).

Patients were hospitalized in the Endocrinology and Diabetes Unit, a tertiary-level DiFoSe, at the “Policlinico Tor Vergata” University Hospital in Rome, Italy. All patients were managed by a multidisciplinary diabetic foot team (MDFT) according to a standardized limb salvage protocol based on the International Working Group on the Diabetic Foot (IWGDF) guidelines [13]. The protocol included prompt lower limb revascularization in patients with chronic limb-threatening ischaemia (CLTI); empirical broad-spectrum antibiotic therapy in the presence of infection, selected according to the clinical presentation and subsequently adjusted based on microbiological results, when available; surgical management tailored to each specific clinical case (e.g., debridement of infected tissue, necrosectomy, minor amputation, bone resection, negative pressure wound therapy, and dermal substitute application); dedicated off-loading; appropriate wound care; and optimization of diabetes management and associated comorbidities.

According to the presence of heart failure, patients were stratified into two groups: those with HF (HF+) and those without HF (HF−). HF status was retrospectively ascertained from the clinical documentation available at the end of hospitalization. HF was defined primarily on the basis of a medically documented clinical history or clinical diagnosis of HF, with N-terminal pro-B-type natriuretic peptide (NT-proBNP) levels and evidence of cardiogenic pulmonary congestion on chest radiography considered supportive findings [14,15]. For the primary analysis, HF was considered as a single clinical category, given the absence of systematic echocardiographic data for all included patients. However, as a descriptive sensitivity analysis, HF was further stratified according to left ventricular ejection fraction (LVEF) among patients with HF and available echocardiographic data, using the following categories: HF with reduced ejection fraction (HFrEF, LVEF ≤ 40%), HF with mildly reduced ejection fraction (HFmrEF, LVEF 41–49%), and HF with preserved ejection fraction (HFpEF, LVEF ≥ 50%) [14].

Baseline clinical characteristics, including demographic, anamnestic, and laboratory data, along with wound characteristics, were recorded. One-year follow-up outcomes were assessed using outpatient medical records and, when necessary, through remote assessment.

### 2.2. Clinical Assessment

Baseline characteristics included age, sex, diabetes type, diabetes duration, DFU history, obesity (defined as a body mass index [BMI] > 30 kg/m^2^), and inability to walk without assistance.

Macrovascular complications and cardiovascular comorbidities were classified as follows. Hypercholesterolemia was defined as ongoing statin therapy or low-density lipoprotein cholesterol (LDL-C) levels >55 mg/dL requiring statin therapy in the setting of very high cardiovascular risk [16]. Hypertension was defined as ongoing antihypertensive therapy or blood pressure ≥140/90 mmHg. Ischaemic heart disease was defined by a history of acute coronary syndrome or coronary revascularization, documented angina, or significant ischaemic changes on electrocardiography. Atrial fibrillation was considered in the case of a documented history of atrial fibrillation, electrocardiographic evidence of atrial fibrillation, or ongoing anticoagulant therapy for atrial fibrillation. Cerebrovascular disease was defined as a history of cerebral ischaemia, previous carotid artery revascularization, or newly diagnosed atherosclerotic plaque with carotid artery stenosis >70%.

Microvascular complications were classified as follows. Diabetic retinopathy was defined as a documented history of diabetic retinopathy or ongoing treatment, including laser therapy or intravitreal injections. End-stage renal disease (ESRD) was defined as the need for chronic dialysis due to diabetic nephropathy.

Additionally chronic respiratory diseases (CRDs) were defined as a documented history of or ongoing therapy for asthma or chronic obstructive pulmonary disease (COPD).

Moreover, baseline laboratory data included metabolic, nutritional, renal, and inflammatory parameters.

### 2.3. Wound Assessment

At the initial assessment, wound characteristics were recorded according to IWGDF definitions [17]. DFU location was recorded according to the anatomical region of the foot, specifically distinguishing between rearfoot and midfoot/forefoot involvement. DFUs were classified as ischaemic/neuro-ischaemic or neuropathic wounds based on a specific neurovascular assessment evaluating the presence of peripheral neuropathy and PAD.

Peripheral neuropathy was diagnosed in the presence of loss of protective sensation (LOPS), assessed by pressure perception using a 10-g Semmes–Weinstein monofilament, and/or reduced vibration perception using a 128-Hz tuning fork or biothesiometer [18].

Peripheral arterial disease (PAD) was suspected based on a history of intermittent claudication and/or previous lower-limb revascularization, abnormal pedal pulses, or, when available, an ankle–brachial index (ABI) < 0.9 or transcutaneous oxygen pressure (TcPO_2_) < 30 mmHg [19]. All patients with suspected PAD underwent ultrasound duplex scanning to confirm the diagnosis, evaluate the morphology and haemodynamic characteristics of the lower-limb vascular tree, and assess the need for and type of lower-limb revascularization. Computed tomography (CT) or magnetic resonance imaging (MRI) was used only when there was uncertainty regarding vascular plaques or the type of intervention required. CLTI was defined as the presence of PAD in combination with gangrene or lower limb ulceration lasting more than two weeks [20].

Diabetic foot infection (DFI) was diagnosed based on the presence of clinical signs of infection, including redness, warmth, swelling, induration, tenderness, pain, and purulent secretion [21]. Only moderate or severe DFIs requiring hospitalization were included in the present study. Diabetic foot osteomyelitis (DFO) was suspected in the presence of a deep ulcer involving the bone, as indicated by exposed bone or a positive probe-to-bone test, and confirmed by radiological imaging, including X-ray or MRI. In most cases, bone biopsy was performed during surgery to identify the causative microorganisms and guide targeted antimicrobial therapy [21].

### 2.4. Treatment of Chronic Limb-Threatening Ischaemia (CLTI)

In patients with neuro-ischaemic DFUs and CLTI, lower-limb revascularization was performed when deemed clinically appropriate, with an overwhelming predominance of endovascular procedures, particularly percutaneous transluminal angioplasty (PTA), in accordance with the established practice and expertise of the MDFT. The revascularization strategy aimed to restore patency of all occluded arteries or, when technically not feasible, of at least the target artery supplying the wound-related angiosome. Patients received dual antiplatelet therapy with acetylsalicylic acid (100 mg daily) and clopidogrel (75 mg daily) before the procedure and for at least 1 month thereafter, extended to 6 months when vascular stenting was performed [22]. Unsuccessful revascularization was defined as the absence of any patent below-the-ankle vessel on final angiography or as indirect revascularization with persistent ischaemia of the wound-related angiosome, documented by TcPO_2_ values < 30 mmHg. In cases of unsuccessful revascularization, autologous peripheral blood mononuclear cells (PB-MNCs) were usually administered as adjuvant therapy [23,24].

### 2.5. One-Year Follow-Up

At discharge, medical therapy was recorded, with particular focus on antidiabetic treatment. During follow-up, patients were regularly assessed in the outpatient setting every 2–4 weeks during the first 6 months and thereafter according to individual clinical needs. Each visit included wound monitoring and management (debridement, dressings and antibiotic therapy, if necessary), as well as off-loading assessment.

In the case of wound healing, patients were referred to a secondary prevention protocol, including regular foot examinations and podiatric care, prescription or renewal of custom-made insoles and appropriate therapeutic footwear, and foot care education for patients, relatives, and caregivers.

Remote monitoring was used only for patients who did not attend scheduled outpatient visits.

### 2.6. Outcomes Measures

After twelve months, the following outcomes were evaluated: wound healing, major amputation, non-fatal major adverse limb and cardiovascular events (NF-MALCE), and all-cause mortality.

Wound healing was defined as complete epithelialization of the wound without discharge, maintained for at least two weeks. Major amputation was defined as any amputation above the ankle. The NF-MALCE endpoint was defined based on the traditional three-point major adverse cardiovascular events (MACE) definition [25], excluding cardiovascular death and including non-fatal myocardial infarction (NF-MI) and non-fatal stroke (NF-stroke), with the addition of CLTI recurrence requiring revascularization. Cause-specific mortality was not evaluated because data on the causes of death were often not available.

The outcomes for the two groups (HF+ vs. HF−) were reported and compared.

### 2.7. Statistical Analysis

All data were collected in a local database and retrospectively analyzed using SAS software version 9.4 (JMP 18; SAS Institute, Cary, NC, USA), which was used as the primary statistical software for data management and the main statistical analyses. ChatGPT (OpenAI, San Francisco, CA, USA; GPT-6) was used as a computational support tool for selected complex or exploratory analyses, including assistance with the development and refinement of statistical code and analytical procedures. Missing data were handled using an available-case analysis, with statistical analyses performed using the available observations for each variable. Continuous variables were expressed as median (interquartile range [IQR]) and compared using the Mann–Whitney U test, given the non-normal distribution of the continuous variables as assessed by the Shapiro–Wilk test. Categorical variables were expressed as numbers and percentages and compared using Fisher’s exact test for binary variables or the chi-square test for variables with more than two categories. A two-sided *p*-value < 0.05 was considered statistically significant.

An exploratory multivariable logistic regression analysis was performed to identify factors independently associated with the presence of HF, entered as the dependent variable. Continuous variables were standardized and expressed as z-scores, whereas NT-proBNP and urinary albumin-to-creatinine ratio (ACR) were first log-transformed using log(1 + x) to account for their skewed distributions and subsequently standardized.

Separate multivariable logistic regression models were subsequently performed for each clinical outcome of interest, with HF as the primary exposure. Models were adjusted a priori for clinically relevant potential confounders, selected based on their established clinical relevance and potential association with both HF and clinical outcomes, including age, sex, ischaemic heart disease, atrial fibrillation, dialysis, haemoglobin, albumin, foot infection and PAD. A subgroup analysis was additionally performed in patients with PAD to assess the consistency of the associations between HF and the clinical outcomes in this higher-risk population. Adjusted odds ratios (aORs) with corresponding 95% confidence intervals (CIs) were reported to quantify the strength of associations.

## 3. Results

### 3.1. Baseline Patient Characteristics According to HF Status

A total of 300 patients were initially selected for the study; of these, 30 were lost to follow-up at one year due to inability to contact or missing the one-year clinical assessment, resulting in a final study population of 270 patients. The median age was approximately 71 years, and most patients were male (74.4%). The majority had type 2 diabetes (95.1%), with a median diabetes duration of 20 years and a median glycated hemoglobin (HbA1c) level of 59 mmol/mol (7.5%). PAD was present in 199 patients (73.7%), and most patients (64.3%) had a previous history of DFU. Overall, 104 patients (38.5%) had HF (HF+), whereas 166 (61.5%) did not (HF−); among patients with PAD, HF was present in 88 cases (44.2%) (Table 2, Table 3 and Table 4).

Patients with HF were older (74 vs. 67 years) and more frequently unable to walk without assistance (28.6% vs. 9.9%); they also had higher rates of ischaemic heart disease (59.6% vs. 27.1%), atrial fibrillation (28.8% vs. 9.0%), and chronic respiratory diseases (26.0% vs. 12.0%) than those without HF (Table 2). Moreover, at baseline laboratory assessment, patients with HF showed higher NT-proBNP levels (315 vs. 95 pg/mL), together with lower albumin (3.4 vs. 3.6 g/dL), estimated glomerular filtration rate (eGFR) (47 vs. 75 mL/min/1.73 m^2^), and haemoglobin levels (11.1 vs. 11.7 g/dL), and higher spot urinary ACR (113 vs. 38 mg/g) and uric acid levels (5.3 vs. 4.6 mg/dL) (Table 3). Regarding baseline wound characteristics, a previous history of DFU (72.7% vs. 59.2%) and PAD (84.6% vs. 66.9%) were more common among patients with HF than among those without HF, whereas no significant differences were observed in ulcer location (rearfoot vs. midfoot/forefoot), infection, osteomyelitis, or gangrene. (Table 4).

### 3.2. Factors Independently Associated with HF

Among the 104 patients with HF, LVEF data were available for 46 (44.2%), allowing descriptive classification by HF phenotype: 15 (32.6%) had HFpEF, 7 (15.2%) had HFmrEF, and 24 (52.2%) had HFrEF.

A multivariable logistic regression analysis was performed to identify factors independently associated with HF. Ischaemic heart disease was strongly associated with HF (aOR 4.45, 95% CI 1.96–10.11; *p* < 0.001), as were higher NT-proBNP levels (aOR 2.78, 95% CI 1.65–4.66; *p* < 0.001). Conversely, higher eGFR was associated with a lower likelihood of HF (aOR 0.56, 95% CI 0.33–0.95; *p* = 0.033) (Supplementary Table S1).

### 3.3. One-Year Outcomes and Multivariable Logistic Regression Analysis

Antidiabetic treatment patterns at discharge differed between patients with and without HF only with respect to the use of basal-bolus insulin (58.7% vs. 44.6%; *p* = 0.033) and metformin (24.0% vs. 45.8%; *p* < 0.001), consistent with the greater cardiorenal complexity of patients with HF. Notably, no significant differences were observed in the use of newer glucose-lowering agents, including glucagon-like peptide-1 (GLP-1) receptor agonists, sodium-glucose cotransporter-2 (SGLT2) inhibitors, and dipeptidyl peptidase-4 (DPP-4) inhibitors. Similarly, lipid-lowering therapy did not differ significantly between groups (Supplementary Table S2).

Regarding HF-related medications, all patients with HF were either already under cardiological follow-up or were referred for cardiological follow-up at discharge, receiving guideline-directed HF therapy recommended whenever compatible with their clinical conditions. In particular, beta-blockers were used in 75.7%; renin–angiotensin–aldosterone system (RAAS)-modulating therapies, including angiotensin-converting enzyme inhibitors (ACEi), angiotensin receptor blockers (ARBs), and angiotensin receptor–neprilysin inhibitors (ARNIs), in 66.7%; mineralocorticoid receptor antagonists (MRAs) in 28.4%; loop diuretics in 80.6%; and SGLT2 inhibitors in 37.5%.

With regard to CLTI treatment, revascularization was performed in 154/199 patients (77.4%), including 64/88 (72.7%) patients with HF and 90/111 (81.1%) patients without HF. PTA failure occurred in 12/154 (7.8%) procedures, including 4/64 (6.2%) in patients with HF and 8/90 (8.9%) in those without HF (*p* = 0.762). Among patients experiencing PTA failure, 8/12 (66.7%) received PB-MNC therapy, including 3/4 (75.0%) patients with HF and 5/8 (62.5%) without HF. 

At one-year follow-up, 180 patients (66.7%) achieved wound healing, 16 (5.9%) underwent major amputation, 76 (28.1%) experienced an NF-MALCE, including 12 (4.4%) NF-MI events, 6 (2.2%) NF-strokes, 73 (36.7%) cases of CLTI recurrence, and 34 (12.6%) patients died (Table 5).

Patients with HF reported poorer outcomes, with lower rates of wound healing (58.6% vs. 71.7%, *p* = 0.033) and higher rates of NF-MALCE (43.3% vs. 18.7%, *p* < 0.001), mainly driven by recurrent CLTI requiring lower-limb revascularization (48.9% vs. 27.0%, *p* < 0.001), as well as higher all-cause mortality (21.1% vs. 7.2%, *p* = 0.001), compared with those without HF (Table 5). Additionally, a descriptive sensitivity analysis stratified the 46/104 (44.2%) patients with HF and available echocardiographic data according to HF phenotype (HFrEF, HFmrEF, and HFpEF), as reported in Supplementary Table S3. Moreover, univariate analyses were performed to explore the associations between HF-related medications at discharge and one-year clinical outcomes among patients with HF (Supplementary Table S4).

Multivariable logistic regression analyses were performed to assess the association between HF and each clinical outcome of interest. NF-MI and NF-stroke were not independently analyzed because of the low number of events. HF was considered the primary exposure, and models were adjusted a priori for clinically relevant potential confounders, including age, sex, ischaemic heart disease, atrial fibrillation, dialysis, haemoglobin, albumin, foot infection and PAD. HF was independently associated with NF-MALCE (aOR 2.90, 95% CI 1.38–6.09; *p* = 0.005), particularly with CLTI recurrence (aOR 2.43, 95% CI 1.11–5.32; *p* = 0.026), while no independent association was observed with healing or mortality (Table 6).

### 3.4. Subgroup Analysis of Patients with PAD

**Figure 1:**
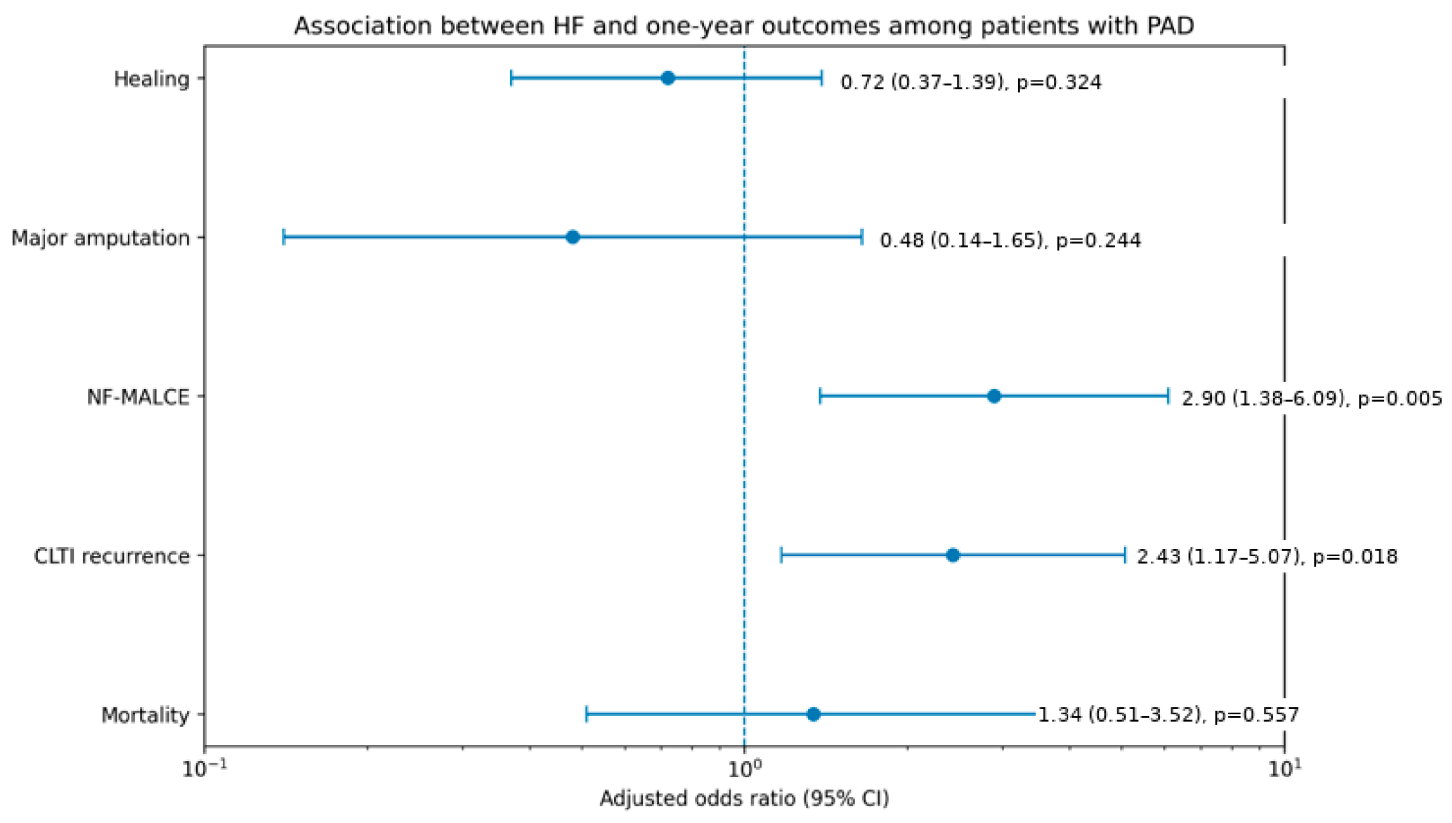
**Multivariable logistic regression analyses of the association between HF and one-year outcomes among patients with PAD.** Forest plot showing adjusted odds ratios (aORs) and 95% confidence intervals (CIs) for the association of interest. Models were adjusted a priori for age, sex, ischaemic heart disease, atrial fibrillation, dialysis, haemoglobin, albumin, and foot infection. HF: heart failure; PAD: peripheral arterial disease; NF-MALCE: non-fatal major adverse limb and cardiovascular events; CLTI: chronic limb-threatening ischaemia.

Given that NF-MALCE was mainly driven by CLTI recurrence, a subgroup analysis restricted to patients with PAD was performed to assess the association between HF and clinical outcomes in the population at risk for CLTI recurrence, thereby providing a more clinically relevant estimate of this association. In the subgroup of patients with PAD, HF remained independently associated with both NF-MALCE (aOR 2.90, 95% CI 1.38–6.09; *p* = 0.005) and CLTI recurrence (aOR 2.43, 95% CI 1.17–5.07; *p* = 0.018). No significant associations were observed for wound healing, major amputation, or mortality (Figure 1).

Notably, dialysis was the only factor independently associated with NF-MALCE in addition to HF (aOR 3.80, 95% CI 1.17–12.40; *p* = 0.027), whereas HF was the only variable independently associated with CLTI recurrence (Supplementary Tables S5 and S6).

## 4. Discussion

The current study shows that HF is highly prevalent among patients hospitalized for DFU and is associated with adverse one-year outcomes, particularly recurrent limb ischaemia requiring revascularization.

In a Swedish study, 76% of patients followed for chronic DFUs showed signs of cardiac dysfunction on echocardiography [26]. In a Chinese study specifically including patients hospitalized for DFUs, HF was present in 64.3% of cases, with its prevalence increasing according to DFU severity as defined by the Wagner classification (from 42.4% to 87.0%) [27]. In the present study, HF was present in 38.5% of patients admitted for DFUs, increasing to 44.2% among those with PAD, which is consistent with a previous study including only hospitalized patients with neuro-ischaemic DFUs, in whom HF was reported in 33.8% of cases [12].

HF appears to identify a particularly vulnerable cardiorenal phenotype among patients hospitalized for DFUs, who already represent a high-risk population [28]. In the current study, patients with HF were older, had reduced mobility, and showed a higher burden of cardiovascular comorbidities, including ischaemic heart disease, atrial fibrillation, and PAD. HF was also associated with a greater prevalence of chronic respiratory disease, poorer renal function, and worse nutritional status. Consistently, in the multivariable analysis, ischaemic heart disease was independently associated with HF, whereas higher eGFR was associated with a lower likelihood of HF, further supporting the close interplay between cardiovascular and renal dysfunction also in this population.

Regarding the outcomes of interest, HF was associated with lower rates of healing (58.6% vs. 71.7%, *p* = 0.033) and higher all-cause mortality (21.1% vs. 7.2%, *p* = 0.001) at the univariate analysis. However, these associations did not remain significant after multivariable adjustment, with HF not being independently associated with either healing or mortality. Similarly, in the high-risk subgroup of patients with PAD, HF was not independently associated with healing or mortality.

The EURODIALE study [8] reported only a borderline association between HF and non-healing at 1 year after multivariable adjustment (OR 1.55, 95% CI 0.99–2.43; *p* = 0.054), whereas Apelqvist et al. [10] reported that the absence of congestive HF was independently associated with a higher likelihood of primary healing (OR 1.81, 95% CI 1.26–2.95; *p* = 0.01) in a large cohort of patients with ischaemic or neuro-ischaemic DFUs and CLTI requiring revascularization. In a more recent study, Rhou et al. [9] reported an independent association between congestive HF and impaired healing (*p* = 0.019) after adjustment for covariates among patients with DFUs treated in an outpatient setting.

In a previous study by Meloni et al. [11], including a larger cohort of both inpatients and outpatients, HF emerged as an independent predictor of one-year mortality (OR 6.4, 95% CI 2.1–14.5; *p* = 0.0001). In another study by the same research group [12], conducted in a more selected cohort of hospitalized patients with ischaemic DFUs and CLTI, HF remained an independent predictor of death at one year after multivariable adjustment (HR 3.6, 95% CI 1.8–6.6; *p* = 0.0001), in a population also characterized by a higher prevalence of dialysis.

The lack of an independent association between HF and healing or mortality in this study, compared with previous reports, may be partly explained by differences in population characteristics, patient selection, and study period. Although speculative, the greater availability and use of newer glucose-lowering therapies with established cardio-renal protective effects may have contributed to the more favourable outcomes observed in this population, particularly regarding mortality. Indeed, differences in glucose-lowering therapy at discharge were mainly observed for traditional agents, whereas the use of newer glucose-lowering therapies (SGLT2 inhibitors and GLP-1 receptor agonists) with established cardiorenal benefits was comparable between groups. Furthermore, in an exploratory analysis of HF-related medications among patients with HF, beta-blocker use was associated with lower odds of healing (OR 0.20, *p* = 0.004), whereas ACEi/ARB/ARNI use and SGLT2 inhibitor use were associated with lower all-cause mortality (OR 0.28, *p* = 0.018, and OR 0.29, *p* = 0.046, respectively). These findings should be interpreted cautiously given the limited sample size, the relatively limited number of events (particularly deaths), and the absence of a specific stratified or interaction analysis according to SGLT2 inhibitor. Moreover, the potential impact of HF severity on these outcomes was not assessed, and residual confounding related to differences in HF severity cannot be excluded.

NF-MALCE occurred in 76 patients (28.1%), with a significantly higher incidence in patients with HF than in those without HF (43.3% vs. 18.7%, respectively; *p* < 0.001). This difference was mainly driven by CLTI recurrence (48.9% vs. 27.0%, respectively; *p* < 0.001). In contrast, no significant differences were observed for NF-MI or NF-stroke.

In the multivariable analysis, HF remained independently associated with NF-MALCE (aOR 2.90, 95% CI 1.38–6.09; *p* = 0.005) and with CLTI recurrence requiring revascularization (aOR 2.43, 95% CI 1.11–5.32; *p* = 0.026). Notably, this association was confirmed in the subgroup of patients with PAD, in whom HF remained significantly associated with NF-MALCE and CLTI recurrence. Importantly, in the multivariable analysis of CLTI recurrence among patients with PAD, HF was the only independent predictor of this outcome (aOR 2.43, 95% CI 1.17–5.07; *p* = 0.018), whereas none of the other covariates included in the model reached statistical significance. This finding further supports an effect of HF that extends beyond the presence of PAD itself and may involve additional pathophysiological mechanisms related to HF.

Several pathophysiological mechanisms may explain the association between HF and cardiovascular and limb-related events. In particular, HF and PAD share several risk factors, pathophysiological mechanisms, and prognostic features, suggesting that the coexistence of these conditions may further increase the vulnerability of patients with PAD to adverse clinical outcomes [29]. HF may adversely affect both systemic peripheral perfusion and microcirculation, creating a milieu that favors recurrent limb ischaemia and limb-related events. First, reduced cardiac output in HFrEF or HFmrEF may impair peripheral perfusion, while venous congestion and peripheral edema can increase interstitial pressure, compromise capillary flow, and impair oxygen diffusion [30]. Conversely, in HFpEF, increased pulsatile load and arterial stiffness may further impair peripheral and microvascular perfusion, thereby disrupting microcirculatory autoregulation [31]. In particular, endothelial dysfunction and chronic neurohormonal and inflammatory activation may impair vasodilatory responses and microcirculatory reserve, further compromising peripheral tissue perfusion [32]. Finally, the frequent coexistence of renal dysfunction, anemia, and malnutrition in patients with HF, as reflected by the clinical phenotype observed in this study, may contribute to a broader state of ‘cardio-renal-metabolic-foot’ vulnerability, potentially increasing susceptibility to limb-related complications [33].

Notably, to the best of our knowledge, no previous study has specifically investigated cardiovascular and limb-related outcomes among patients hospitalized for DFUs according to the presence of HF. In addition, the sample size enabled comprehensive multivariable analyses and subgroup assessments, strengthening the robustness of the findings. In patients hospitalized for DFUs, HF identifies a particularly high-risk cardiorenal phenotype and is independently associated with limb-related adverse events, particularly recurrent limb ischaemia requiring revascularization. These results support regular foot screening and closer vascular surveillance, together with an integrated multidisciplinary approach including optimization of HF management and coordinated cardio-renal-metabolic and nutritional care, with potential implications for more proactive clinical management of these high-risk patients.

The present study has several limitations.

First, its retrospective, single-center design and inclusion of hospitalized patients from a tertiary DiFoSe may limit the generalizability of the findings to the broader population of patients with DFUs. However, all patients were treated within the same specialized setting and by the same MDFT, with no relevant differences in the overall clinical management of patients with and without HF, including the type of antidiabetic therapy.

Second, the relatively low number of events for some outcomes limited multivariable analyses of NF-MI and NF-stroke.

Third, PAD severity was not stratified, limiting the assessment of recurrent CLTI according to PAD severity, while recurrent CLTI was considered only in patients with non-healing wounds requiring repeat revascularization; therefore, ischaemic recurrences occurring after wound healing cannot be excluded. In addition, detailed angiographic data on the extent and target of revascularization were not systematically available, and the small number of PTA failures and PB-MNC treatments precluded meaningful outcome analyses, especially regarding CLTI recurrence.

Fourth, the lack of systematic echocardiographic assessment, including left ventricular ejection fraction (LVEF), precluded reliable classification of HF into HFrEF, HFmrEF, and HFpEF and limited the objective characterization of HF severity. Although a purely descriptive sensitivity analysis was performed in the subset with available echocardiographic data, the limited availability of LVEF data precluded a more robust phenotype-specific analysis. Moreover, the absence of consistent information on functional status, including New York Heart Association (NYHA) class [15], further limited the assessment of HF severity and its potential relationship with clinical outcomes.

Fifth, data on the clinical evolution of HF during follow-up, including hospitalizations for HF decompensation, changes in functional status, and longitudinal trends in natriuretic peptide levels, were not collected. Consequently, the potential impact of changes in HF severity during follow-up on limb-related outcomes could not be evaluated.

Finally, the observational design does not allow causal inferences, and residual confounding cannot be excluded despite multivariable adjustment. Moreover, measures of model performance were not performed, as they were not considered strictly necessary for the specific purpose of the study, which was to estimate and interpret adjusted odds ratios.

## 5. Conclusions

This study reinforces the concept that diabetic foot disease is not simply a local wound problem, but a systemic syndrome in which HF may play a key role. Therefore, HF should be systematically considered in patients hospitalized for DFUs, as its presence identifies a high-risk cardiorenal phenotype and is independently associated with an increased risk of limb-related adverse events. Accordingly, patients with HF warrants dedicated cardiovascular assessment within a multidisciplinary, patient-centred approach. Future prospective studies are needed to confirm these findings, clarify the underlying mechanisms, and determine their clinical implications.

## Figures and Tables

**Table 1:** Eligibility criteria.

Inclusion Criteria	Exclusion Criteria
- Age ≥18 years; - Diabetes mellitus; - Hospitalization for a diabetic foot ulcer complicated by limb ischaemia, infection, or requiring surgical treatment.	- Primary amputation due to lack of anatomical suitability for foot salvage.

**Table 2:** **Baseline clinical characteristics of the overall study population and according to HF status.** Missing data were handled using available-case analysis, with variable-specific denominators. Continuous variables are expressed as median (interquartile range).

Variables	Overall (n = 270)	HF+ (n = 104)	HF− (n = 166)	*p*-Value
Age (years)	71.0 [61.0–77.0]	74.0 [65.8–79.2]	67.0 [59.0–76.0]	<0.001
Sex (male)	201 (74.4%)	73 (70.2%)	128 (77.1%)	0.251
Type 2 diabetes	255 (95.1%)	100 (98.0%)	155 (93.4%)	0.152
Diabetes duration (years)	20.0 [10.0–30.0]	20.0 [15.0–30.0]	20.0 [10.0–30.0]	0.125
Obesity	52 (25.4%)	26 (31.7%)	26 (21.1%)	0.102
Hypercholesterolemia	250 (92.6%)	100 (96.2%)	150 (90.4%)	0.096
Hypertension	232 (85.9%)	92 (88.5%)	140 (84.3%)	0.374
Ischaemic heart disease	107 (39.6%)	62 (59.6%)	45 (27.1%)	<0.001
Atrial fibrillation	45 (16.7%)	30 (28.8%)	15 (9.0%)	<0.001
Cerebrovascular disease	23 (8.5%)	13 (12.5%)	10 (6.0%)	0.075
Diabetic retinopathy	73 (27.7%)	26 (26.3%)	47 (28.5%)	0.777
Dialysis (ESRD)	24 (8.9%)	11 (10.6%)	13 (7.8%)	0.511
CRDs	47 (17.4%)	27 (26.0%)	20 (12.0%)	0.005
Inability to walk	43 (17.2%)	28 (28.6%)	15 (9.9%)	<0.001

HF: heart failure; ESRD: end-stage renal disease; CRDs: chronic respiratory diseases.

**Table 3:** **Baseline laboratory parameters of the overall study population and according to HF status.** Missing data were handled using available-case analysis, with variable-specific denominators. Continuous variables are expressed as median (interquartile range).

Variables	Overall (n = 270)	HF+ (n = 104)	HF− (n = 166)	*p*-Value
HbA1c (mmol/mol)	59.0 [51.0–75.0]	59.0 [51.0–71.0]	59.0 [51.0–77.0]	0.703
Total cholesterol (mg/dL)	122.0 [101.0–153.0]	117.0 [96.0–146.0]	127.0 [102.0–154.5]	0.100
LDL-C (mg/dL)	72.0 [49.0–94.0]	63.5 [45.8–89.5]	76.0 [50.8–95.2]	0.103
HDL-C (mg/dL)	31.0 [24.0–38.0]	30.5 [23.0–38.0]	31.0 [25.0–37.2]	0.808
Triglycerides (mg/dL)	104.0 [83.0–136.0]	106.0 [79.8–134.2]	103.0 [84.0–137.0]	0.956
NT-proBNP (pg/mL)	134.0 [55.8–381.5]	315.0 [141.0–841.0]	95.0 [35.0–173.0]	<0.001
Albumin (g/dL)	3.5 [3.2–3.8]	3.4 [3.1–3.7]	3.6 [3.2–3.9]	0.016
eGFR (mL/min/1.73 m^2^)	65.0 [41.0–84.9]	47.0 [34.0–65.1]	75.0 [51.2–98.0]	<0.001
Spot urinary ACR (mg/g)	54.0 [20.0–199.5]	113.0 [37.0–625.0]	38.0 [16.5–109.0]	<0.001
Uric acid (mg/dL)	4.8 [3.7–6.0]	5.3 [4.0–6.7]	4.6 [3.6–5.6]	<0.001
Haemoglobin (g/dL)	11.4 [10.2–12.8]	11.1 [9.8–12.4]	11.7 [10.4–13.1]	0.012
WBC (cells/mcl)	8.4 [6.6–11.1]	8.0 [6.7–11.4]	8.9 [6.5–10.8]	0.665
CRP (mg/L)	32.0 [7.9–92.0]	34.4 [14.0–89.3]	29.8 [6.8–105.1]	0.774

HF: heart failure; HbA1c: glycated hemoglobin; LDL-C: low-density lipoprotein cholesterol; HDL-C: high-density lipoprotein cholesterol; NT-proBNP: N-terminal pro-B-type natriuretic peptide; eGFR: estimated glomerular filtration rate; ACR: albumin-to-creatinine ratio; WBC: white blood cells; CRP: C-reactive protein.

**Table 4:** **Baseline wound characteristics of the overall study population and according to HF status.** Missing data were handled using available-case analysis, with variable-specific denominators.

Variables	Overall (n = 270)	HF+ (n = 104)	HF− (n = 166)	*p*-Value
Rearfoot localization	35 (13.0%)	19 (18.3%)	16 (9.6%)	0.061
PAD	199 (73.7%)	88 (84.6%)	111 (66.9%)	0.002
Gangrene	149 (55.2%)	62 (59.6%)	87 (52.4%)	0.260
Infection	215 (79.6%)	78 (75.0%)	137 (82.5%)	0.162
Osteomyelitis	111 (41.1%)	37 (35.6%)	74 (44.6%)	0.163
DFU history	148 (64.3%)	64 (72.7%)	84 (59.2%)	0.047

HF: heart failure; PAD: peripheral arterial disease; DFU: diabetic foot ulcer.

**Table 5:** **One-year follow-up outcomes in the overall study population and according to HF status.** CLTI recurrence denominator restricted to patients with peripheral arterial disease.

Outcomes	Overall (n = 270)	HF+ (n = 104)	HF− (n = 166)	*p*-Value
Healing	180 (66.7%)	61 (58.6%)	119 (71.7%)	0.033
Major amputation	16 (5.9%)	6 (5.8%)	10 (6.0%)	1.0
NF-MALCE	76 (28.1%)	45 (43.3%)	31 (18.7%)	<0.001
NF-MI	12 (4.4%)	6 (5.8%)	6 (3.6%)	0.688
NF-stroke	6 (2.2%)	4 (3.8%)	2 (1.2%)	0.565
CLTI recurrence	73/199 (36.7%)	43/88 (48.9%)	30/111 (27.0%)	<0.001
Mortality	34 (12.6%)	22 (21.1%)	12 (7.2%)	0.001

HF: heart failure; NF-MALCE: non-fatal major adverse limb and cardiovascular events, including non-fatal myocardial infarction (NF-MI), non-fatal (NF) stroke, and chronic limb-threatening ischaemia (CLTI) recurrence.

**Table 6:** **Multivariable logistic regression analyses of the association between HF and one-year outcomes in the overall study population.** Results are reported as adjusted odds ratios (aORs) with 95% confidence intervals (CIs). Models were adjusted a priori for clinically relevant potential confounders, including age, sex, ischaemic heart disease, atrial fibrillation, dialysis, haemoglobin, albumin, foot infection and peripheral arterial disease.

Outcomes	aOR HF (95% CI)	*p*-Value
Healing	0.65 (0.36–1.20)	0.167
Major amputation	0.61 (0.19–1.93)	0.399
NF-MALCE	2.90 (1.38–6.09)	0.005
CLTI recurrence	2.43 (1.11–5.32)	0.026
Mortality	1.71 (0.70–4.19)	0.243

HF: heart failure; NF-MALCE: non-fatal major adverse limb and cardiovascular events; CLTI: chronic limb-threatening ischaemia.

## Data Availability

The original contributions presented in this study are included in the article. Further inquiries regarding the data may be directed to the corresponding author.

## Supplementary Materials

The following supporting information can be downloaded at: https://www.mdpi.com/article/10.3390/jcm15208024/s1, Table S1: Exploratory multivariable logistic regression analysis of factors associated with HF; Table S2: Medications at discharge in the overall study population and according to HF status; Table S3: Descriptive sensitivity analysis of one-year outcomes according to HF phenotype among the patients with HF and available echocardiographic data; Table S4: Univariate analysis of the associations between HF-related medications at discharge and one-year outcomes among patients with HF; Table S5: Multivariable logistic regression analysis of one-year NF-MALCE outcome among patients with PAD; Table S6: Multivariable logistic regression analysis of one-year CLTI recurrence outcome among patients with PAD.
