# Supplementary material for: Heart Failure Is Associated with Limb-Related Adverse Events in Patients Hospitalized for Diabetic Foot Ulcers
Source: J Clin Med. 2026 Oct 16;15(20):8024. doi: 10.3390/jcm15208024 (PMC13706131; doi:10.3390/jcm15208024)
Supplement: Supplementary file 1 [file jcm-15-08024-s001.zip › jcm-4558254-supplementary.pdf]

Supplementary Table S1. Exploratory multivariable logistic regression analysis of factors associated with HF. HF was the dependent variable. Continuous variables were standardized per 1 standard deviation (SD); NT-proBNP and ACR were log-transformed using log(1+x). Results are reported as adjusted odds ratios (aORs) with 95% confidence intervals (CIs).

Predictor	aOR	95%CI	*p*-Value
Age, per 1 SD	1.04	0.66–1.65	0.863
Sex (male)	0.65	0.27–1.56	0.335
PAD	1.83	0.70–4.77	0.215
Ischaemic heart disease	4.45	1.96–10.11	<0.001
Atrial fibrillation	2.60	0.92–7.32	0.071
log NT-proBNP, per 1 SD	2.78	1.65–4.66	<0.001
Albumin, per 1 SD	0.24	0.04–1.47	0.122
eGFR, per 1 SD	0.56	0.33–0.95	0.033
log ACR, per 1 SD	1.38	0.89–2.12	0.146
Uric acid, per 1 SD	1.34	0.82–2.18	0.247
Haemoglobin, per 1 SD	0.94	0.51–1.73	0.851

HF: heart failure; PAD: peripheral arterial disease; NT-proBNP: N-terminal pro-B-type natriuretic peptide; eGFR: estimated glomerular filtration rate; ACR: albumin-to-creatinine ratio.

Supplementary Table S2. Medications at discharge in the overall study population and according to HF status. Missing data were handled using available-case analysis, with variable-specific denominators.

Variables	Overall (n = 270)	HF+ (n = 104)	HF− (n = 166)	*p*-Value
Basal insulin	60 (22.2%)	24 (23.1%)	36 (21.7%)	0.881
Basal-bolus insulin	135 (50.0%)	61 (58.7%)	74 (44.6%)	0.033
Metformin	101 (37.4%)	25 (24.0%)	76 (45.8%)	<0.001
GLP-1 receptor agonists	65 (24.1%)	21 (20.2%)	44 (26.5%)	0.247
SGLT2 inhibitors	100 (37.0%)	39 (37.5%)	61 (36.7%)	0.898
DPP-4 inhibitors	20 (7.4%)	10 (9.6%)	10 (6.0%)	0.340
Lipid-lowering therapy	239 (88.5%)	95 (91.3%)	144 (86.7%)	0.327

HF: heart failure; GLP-1: glucagon-like peptide-1; SGLT2: sodium-glucose cotransporter-2; DPP-4: dipeptidyl peptidase-4.

Supplementary Table S3. Descriptive sensitivity analysis of one-year outcomes according to HF phenotype among the patients with HF and available echocardiographic data. Results are reported as numbers and percentages in the 46/104 (44.2%) patients included in this analysis.

Outcomes	**HFpEF** (n = 15, 32.6%)	**HFmrEF** (n = 7, 15.2%)	**HFrEF** (n = 24, 52.2%)
Healing	8 (53.3%)	5 (71.4%)	16 (66.7%)
Major amputation	1 (6.7%)	0 (0%)	2 (8.3%)
NF-MALCE	9 (60.0%)	1 (14.3%)	14 (58.3%)
NF-MI	0 (0%)	0 (0%)	3 (12.5%)
NF-stroke	0 (0%)	0 (0%)	2 (8.3%)
CLTI recurrence	9 (60.0%)	1 (14.3%)	13 (54.2%)
Mortality	1 (6.7%)	1 (14.3%)	7 (29.2%)

HF: heart failure; HFpEF: heart failure with preserved ejection fraction; HFmrEF: heart failure with mildly reduced ejection fraction; HFrEF: heart failure with reduced ejection fraction; NF-MALCE: non-fatal major adverse limb and cardiovascular events, including non-fatal myocardial infarction (NF-MI), non-fatal (NF) stroke, and chronic limb-threatening ischaemia (CLTI) recurrence.

Supplementary Table S4. Univariate analysis of the associations between HF-related medications at discharge and one-year outcomes among patients with HF. Results are reported as odds ratios (ORs) with corresponding p-values.

Variables	Beta-blockers (75.7%)	ACEi/ARBs/ARNIs (66.7%)	MRAs (28.4%)	Loop diuretics (80.6%)	SGLT2i (37.5%)
Healing	0.20 (0.004)	1.49 (0.395)	0.93 (1.0)	0.41 (0.131)	1.71 (0.222)
Major amputation	1.67 (1.0)	1.02 (1.0)	0.50 (1.0)	- (0.595)	0.80 (1.0)
NF-MALCE	1.95 (0.223)	0.55 (0.250)	0.80 (0.806)	1.0 (1.0)	0.69 (0.508)
CLTI recurrence	1.71 (0.328)	0.66 (0.478)	0.93 (1.0)	0.88 (1.0)	0.82 (0.822)
Mortality	3.97 (0.091)	0.28 (0.018)	1.61 (0.425)	1.11 (1.0)	0.29 (0.046)

HF: heart failure; ACEi: angiotensin-converting enzyme inhibitors; ARBs: angiotensin receptor blockers; ARNIs: angiotensin receptor–neprilysin inhibitors; MRAs: mineralocorticoid receptor antagonists; SGLT2i: sodium-glucose cotransporter-2 inhibitors; NF-MALCE: non-fatal major adverse limb and cardiovascular events; CLTI: chronic limb-threatening ischaemia.

Supplementary Table S5. Multivariable logistic regression analysis of one-year NF-MALCE outcome among patients with PAD. Age is expressed per 1-year increment; haemoglobin and albumin are expressed per 1 g/dL increment. Results are reported as adjusted odds ratios (aORs) with 95% confidence intervals (CIs).

Variables	aOR for NF-MALCE (95% CI)	*p*-Value
Heart failure	2.90 (1.38–6.09)	0.005
Age (per year)	1.02 (0.99–1.06)	0.229
Sex (male)	2,38 (0,96–5,88)	0.060
Ischaemic heart disease	0.75 (0.36–1.56)	0.441
Atrial fibrillation	1.08 (0.46–2.53)	0.852
Dialysis	3.80 (1.17–12.40)	0.027
Haemoglobin (per 1 g/dl)	0.87 (0.74–1.02)	0.093
Albumin (per 1 g/dl)	2.12 (0.93–4.85)	0.076
Foot infection	0.49 (0.23–1.04)	0.063

NF-MALCE: non-fatal major adverse limb and cardiovascular events; PAD: peripheral arterial disease.

Supplementary Table S6. Multivariable logistic regression analysis of one-year CLTI recurrence outcome among patients with PAD. Age is expressed per 1-year increment; haemoglobin and albumin are expressed per 1 g/dL increment. Results are reported as adjusted odds ratios (aORs) with 95% confidence intervals (CIs).

Variables	aOR for CLTI recurrence (95% CI)	*p*-Value
Heart failure	2.43 (1.17–5.07)	0.018
Age (per year)	1.02 (0.99–1.06)	0.213

Sex (male)	2,33 (0,93–5,88)	0.073
Ischaemic heart disease	0.86 (0.41–1.79)	0.683
Atrial fibrillation	1.53 (0.66–3.53)	0.322
Dialysis	2.34 (0.72–7.61)	0.156
Haemoglobin (per 1 g/dl)	0.85 (0.71–1.02)	0.077
Albumin (per 1 g/dl)	2.16 (0.93–5.03)	0.074
Foot infection	0.55 (0.26–1.18)	0.125

CLTI: chronic limb-threatening ischaemia; PAD: peripheral arterial disease.
